# The Use of Stem Cells to Model Amyotrophic Lateral Sclerosis and Frontotemporal Dementia: From Basic Research to Regenerative Medicine

**DOI:** 10.1155/2016/9279516

**Published:** 2016-02-07

**Authors:** Erin C. Hedges, Vera J. Mehler, Agnes L. Nishimura

**Affiliations:** Department of Basic and Clinical Neuroscience, Institute of Psychiatry, Psychology and Neuroscience (IOPPN), King's College London and King's Health Partners, London SE5 9RX, UK

## Abstract

In recent years several genes have linked amyotrophic lateral sclerosis (ALS) and frontotemporal dementia (FTD) as a spectrum disease; however little is known about what triggers their onset. With the ability to generate patient specific stem cell lines from somatic cells, it is possible to model disease without the need to transfect cells with exogenous DNA. These pluripotent stem cells have opened new avenues for identification of disease phenotypes and their relation to specific molecular pathways. Thus, as never before, compounds with potential applications for regenerative medicine can be specifically tailored in patient derived cultures. In this review, we discuss how patient specific induced pluripotent stem cells (iPSCs) have been used to model ALS and FTD and the most recent drug screening targets for these diseases. We also discuss how an iPSC bank would improve the quality of the available cell lines and how it would increase knowledge about the ALS/FTD disease spectrum.

## 1. Introduction

*(1) ALS and FTD: A Neurodegenerative Disease Spectrum*. Amyotrophic lateral sclerosis (ALS), often referred to as motor neuron disease, is characterised by degeneration of upper and lower motor neurons (MNs) leading to muscle wasting and paralysis. Limb onset is seen in 61.2% of ALS patients, with bulbar onset in 30.1% [1]. Symptoms often include increased muscle tone, hyperreflexia, slowness of movement, fasciculation, and muscle weakening [2]. ALS may be considered a rare disease with a median incidence rate of 2.08 per 100,000 in Europe, but it is suggested that the social and economic burden is substantial [3]. Multiple cellular pathologies and genetic abnormalities have been implicated in ALS, resulting in varying clinical presentation. For example, ALS caused by a mutation in the gene* chromosome 9 open reading frame 72* (*C9ORF72*) has a higher incidence of behavioural and cognitive impairments and comorbidity with frontotemporal dementia (FTD) compared with other ALS-associated genes [4]. Furthermore, a subset of* C9ORF72* ALS cases with no FTD specifically showed p62 inclusions in the cerebral cortex, hippocampus, and cerebellum [5].* Fused in sarcoma* (*FUS*) linked ALS is less likely to be linked with FTD, and a specific* FUS* mutation (R521C) is associated with earlier disease onset and increased likelihood of dropped head syndrome [6], further indicating the clinical variety. Approximately 30 genes have been linked directly to ALS, and 126 genes somewhat related to ALS phenotype have been identified [7]. Approximately 10% of ALS cases are familial (FALS), often with an autosomal dominant inheritance pattern, and the remaining 90% of cases occur sporadically (SALS) [8], many with an unknown cause. No successful treatment strategy exists for ALS, with a median survival time of 3–5 years from diagnosis [9]. The current primary treatment is the antiexcitotoxicity drug riluzole; however the beneficial effects of this are limited to 6 months [10].

Increasingly, evidence suggests that a broad disease spectrum exists encompassing both ALS and FTD. FTD describes a group of progressive dementias characterised by degeneration of the frontotemporal cortex. The FTD phenotype varies but symptoms may include behavioural changes, difficulties with language, and loss of executive function [11]. It is suggested that 34.1–50% of ALS patients present with some cognitive impairment, and 5–15% of patients meet the criteria for FTD [12, 13]. Mutations to multiple genes can cause either FTD or ALS phenotype, indicating that the two classifications are varying manifestations of one disease, including the genes in Table 1. Furthermore, ALS or FTD can occur between generations in some familial cases [14]. Multiple cellular pathologies have been identified in ALS/FTD, which have been extensively reviewed [8, 15], yet the full extent of disease mechanisms remains elusive.

*(2) Modelling ALS/FTD for Research*. Research models aim to represent disease to facilitate understanding of disease mechanisms and as a means for development of treatment strategies, including drug discovery for both preventative and regenerative therapies. Use of stem cell technology for modelling disease is becoming an important tool, and there is an increasing body of ALS/FTD research that has utilized such technologies. Pluripotent stem cells are able to differentiate into almost all cell types from the three germ layers [16], including neurons, motor neurons, and glial cells. Thus, guided differentiation of pluripotent stem cells into a desired cell lineage produces an* in vitro* model representative of cell lineages specifically targeted in disease.

## 2. Lessons from Stem Cell Models

### 2.1. Embryonic Stem Cells

Much of the technology for guided differentiation of pluripotent stem cells comes from research using embryonic stem cells (ESCs). Mouse ESCs were first differentiated into lower motor neurons in 2002 by Wichterle et al. as a result of studying morphogens involved in neurogenesis [28]. This was followed by formation of motor neurons from human ESCs [29]. Ethical concerns with using human ESCs, among other limitations, mean there has been a reduction in ESC based research since the development of induced pluripotent stem cell (iPSC) technology; however some research utilizing ESCs in ALS exists. Human ESC derived glial cells with an inserted* superoxide dismutase* (*SOD1*) mutation were shown to cause toxic effects to cocultured MNs but not interneurons, implicating MNs as specifically sensitive in ALS [30]. Astrocyte mediated toxicity was consequently shown to be related to increased inflammatory response in* SOD1* human ESC derived cultures [31]. More recently mouse ESC derived cultures harbouring the* SOD1* G93A mutation were used to screen small molecules with potential to rescue ALS phenotype [32]. This identified the kinase inhibitor kenpaullone as able to prolong motor neuron survival, an effect that was consequently recapitulated in* SOD1* and* TAR DNA binding protein* (*TARDBP*) ALS patient derived iPSCs, indicating how a combination of stem cell models might aid research.

### 2.2. Induced Pluripotent Stem Cells

iPSCs are pluripotent cells that have been derived from somatic cells through the introduction of at least four key pluripotency genes. This technology was first demonstrated by Takahashi and Yamanaka (2006) in mouse fibroblasts [33], and this was shortly followed by formation of iPSCs from human somatic cells [34]. Since this discovery differentiation of iPSCs into various cell lineages has been successful and some lessons about ALS and FTD have been learned, particularly through utilization of iPSC derived MNs (summarised in Table 2). Much research has alluded to the prospect of iPSC derived neurons as a drug screening tool in ALS/FTD [21, 32, 35].

*SOD1*. The first example of MN formation from ALS patient iPSCs was through utilizing patient fibroblasts with a* SOD1* mutation [36]. More recently iPSCs from* SOD1* ALS patients were used to identify neurofilament aggregation and neurite degeneration in spinal MNs [27]. Mutant* SOD1* was shown to sequester neurofilament subunit mRNA, resulting in less mRNA available for translation and consequently less protein, indicating a novel mechanism for axonal degeneration in* SOD1* MNs.

*TDP-43*. A common proteinopathy seen in ALS/FTD is dysregulated transactive response DNA binding protein 43 kDa (TDP-43). TDP-43 proteinopathy manifests as mislocalization of usually nuclear TDP-43 to the cytoplasm, resulting in both loss of nuclear function and toxic gain of function mechanisms [37, 38]. Mutations to the TDP-43 gene* TARDBP* can cause ALS/FTD [39–41], alluding to the role of TDP-43 dysfunction in disease mechanisms.

iPSC derived MNs from patients with* TARDBP* mutations have increased levels of soluble and detergent-resistant TDP-43 and show decreased cell survival, suggesting this model is representative of ALS pathology [17]. This study found that patient derived cultures show increased vulnerability to antagonism of the cell cycle-regulating PI3K pathway, highlighting how stem cell cultures can be used to uncover disease characteristics. An independent study identified stress induced changes in* TARDBP* ALS patient derived neurons only, including TDP-43 mislocalization, reduced total TDP-43, and decreased levels of microRNA-9 [42]. The reduction in microRNA-9 was seen in patient derived culture from two patients with different* TARDBP* mutations, indicating a potential role of microRNAs in ALS/FTD pathogenesis. Neuronal cultures from* TARDBP* ALS patient iPSCs show disease characteristic TDP-43 aggregates and the same morphological differences seen in animal ALS models, indicating that they are representative of the disease [35]. Furthermore this group showed that patient derived cultures are more vulnerable to arsenite induced stress than controls and exhibit altered gene expression for genes involved in RNA metabolism and cytoskeletal proteins. They also identified a histone acetyltransferase inhibitor as a potential tool for reversing ALS phenotype through drug screening assays. One role of TDP-43 and its functional homologue FUS is processing long pre-mRNAs, a function that may be lost in ALS/FTD, and this mechanism was identified partly in stem cell derived cultures [43].

Glial cells have been implicated in ALS/FTD pathology, and astroglia derived from* TARDBP* ALS patient iPSCs show TDP-43 proteinopathy such as mislocalization, increased total cellular levels, and decreased cell survival [18]. Interestingly these astroglia were shown to have no adverse effects on either wild type or mutation containing iPSC derived neurons in coculture, indicating a cell-autonomous disease mechanism.

MNs in iPSC derived cultures from both* TARDBP* and* C9ORF72* ALS patients showed hyperexcitability followed by loss of action potential output and activity at the synapse. This was associated with a decrease in voltage dependent Na^+^ and K^+^ movement, suggesting reduced or dysfunctional ion channels [52]. The authors suggest that this disruption to ion channel organisation might contribute to the initiation of downstream degenerative pathways, indicating how use of stem cell derived cultures might aid the uncovering of disease mechanisms associated with a preclinical time point.

*C9ORF72*. A hexanucleotide repeat expansion in the first intron of the* C9ORF72* gene manifests as ALS and/or FTD, both familial and sporadic, supporting the notion that ALS and FTD exist within the same disease spectrum [49, 50]. Almeida et al. (2013) were the first group to represent* C9ORF72* expansion mutations in patient derived cultures, showing that iPSC derived neurons harbour typical disease characteristics including the presence of aggregated expansion mRNA into foci and formation of repeat-associated non-ATG (RAN) translation dipeptides [22]. A potential role of impaired autophagy was identified as increased levels of p62 and increased sensitivity to stress caused by autophagy inhibition which were seen in the patient derived cultures. Following this, iPSC derived cultures have been used to uncover* C9ORF72* ALS/FTD characteristics including colocalization of repeat mRNA with RNA binding proteins, altered gene expression patterns, susceptibility to excitotoxicity, and MN dysfunction [23, 24, 54]. Furthermore* C9ORF72* disease characteristics in iPSC derived cultures were mitigated with antisense oligonucleotide intervention [23], further indicating the role of stem cell models in development of treatment strategies. Recent evidence has indicated a role of nucleocytoplasmic transport dysfunction in* C9ORF72* ALS/FTD pathology. Neuronal cultures from patient iPSCs have contributed to identification of nuclear RNA export defects [25] and nuclear import impairment caused by nucleocytoplasmic transport protein mislocalization [26]. 

*SALS*. An important benefit of patient derived tissue culture is the opportunity to model disease when the genetic causes are unknown, such as in SALS cases. Through formation of motor neurons from multiple SALS patient derived iPSCs, Burkhardt et al. (2013) identified TDP-43 aggregates in three SALS cases which were unrelated to genes already known to be implicated in ALS TDP-43 proteinopathy [21]. This aggregation was consequently visualised in postmortem analysis of one of the patients from which the iPSCs had been derived, indicating that iPSC derived culture accurately represents cellular phenotype and that iPSCs are a useful tool for uncovering disease implications when the root cause of an illness is unknown.

*FTD*. Comparison of iPSC derived neurons and glia from patients with sporadic or* progranulin* (*PGRN*) mutation associated FTD highlighted phenotypic differences between groups [19]. Varying cellular levels of progranulin and contrasting sensitivity to various cellular stressors such as kinase inhibition highlighted differences seen between subtypes of FTD. Consequently iPSC derived neurons were used in the validation of small molecules with potential to rescue* PGRN* associated FTD cellular phenotype [53]. Behavioural variant FTD patient derived neuronal cultures exhibit altered levels of AMPA receptors and microRNA-124 [55]. These data, alongside results from animal models and patient autopsy studies, have implicated these cellular changes as related to behavioural symptoms and social dysfunction in particular.

**Figure 1:**
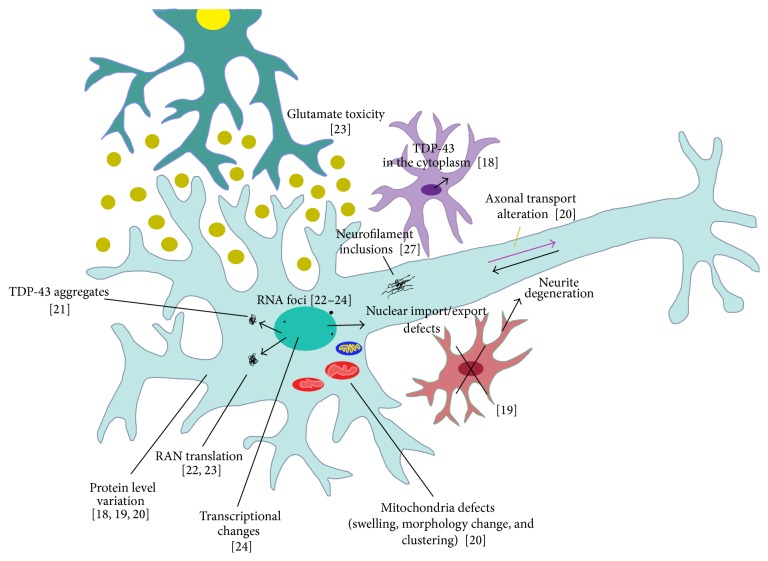
Overview representation of disease pathogenesis of ALS and FTD in cells derived from patient somatic lines. Neurons/motor neurons were differentiated from human induced pluripotent stem cells and varied phenotypes were observed: mutant protein may be mislocalized into a different cellular compartment (TDP-43 [17, 18]); overexpression and downregulation of mutant proteins were observed [18–20]; formation of cellular aggregates [21]; formation of repeat-associated non-ATG (RAN) peptides [22, 23]; formation of RNA foci [22–24]; glutamate toxicity [23]; mitochondria defects [20]; nuclear import/export impairment [25, 26]; formation of neurofilament inclusions [27] and axonal transport defects [20]. In the absence of glia, SOD1 models formed neurofilament inclusions which lead to neurite degeneration in motor neurons [27].

A schematic representation of the impact of stem cell research in modelling ALS/FTD is seen in Figure 1.

## 3. Generation of an iPSC Bank

To date, most iPSC research has been completed utilizing very few patient cell lines, dependent on both the availability of patient samples and generosity of the patients. Until more lines from different patients with the same disease mutation and a large collection of controls are established, it is difficult to ascertain if one particular phenotype corresponds to the disease itself or if it is caused by serendipitous random events. New technologies including genome-editing tools have been used to generate new lines of iPSCs by introducing the desired mutation in specific regions of the genome with minimum off-target effects [56]. What is still unknown is whether various aspects of each individual's genetic background might play an important role in the disease phenotype, a factor that is excluded in genome-edited lines. The generation of a large iPSC bank would circumvent these problems and give researchers access to several patient specific lines.

Previously, human ESC banks have been successfully established with clinical-compliant cell lines suitable for therapeutic use and for preclinical research [57]. The experiences gained in the process of generating human ESC banks should therefore be exploited with regard to enabling more efficient generation of iPSC banks. Additionally, a hypothetical model has been proposed that would allow low-cost and high-quality drug discovery and development using iPSCs [58]. Thus, the current lengthy process of therapeutic discovery including* in vitro* tests, animal models, and clinical trials could be shortened significantly with the establishment of iPSC banks.

### 3.1. The Premise for a Comprehensive Stem Cell Bank

The process of reprogramming somatic cells into iPSCs and subsequent differentiation into a desired cell type requires multiple steps. First, biological material is obtained from patients. Biological material includes hair and blood samples that can be routinely collected from patients in any medical practice or skin biopsies which require a more elaborate collection scheme. Once the patient cells have been expanded in the laboratory they can be reprogrammed into iPSCs using standardized protocols. Selection of iPSC clones and quality control of these lines for the maintenance and provision of large iPSC stocks should be rigorous and obligatory [59]. This should include analyses of DNA (sequencing the samples for genes related to the disease), analysis of number and appearance of chromosomes (karyotyping), collection of several clones from the same individual and selection of similar lines to reduce clonal variation, selection of lines based on appropriate cellular morphology, expression of pluripotent markers, and presence of electrophysiological activity in neuron derived lines [60].

An iPSC bank would provide a great opportunity to increase our knowledge about disease mechanisms and would serve as a platform for drug screening and discovery of new treatments. There are different initiatives, such as CCRM (http://ccrm.ca/), CIRM (https://www.cirm.ca.gov/), HiPSCi (http://www.hipsci.org/), and StemBANCC (http://www.stembancc.org/), which are currently trying to establish comprehensive iPSC libraries [59].

### 3.2. The Role of iPSC Banks in Revealing Pathogenic Mechanisms of ALS/FTD

Stem cell derived models can be utilized to identify pathogenic mechanisms underlying neurodegenerative disorders. Here, ALS and FTD are used as an example to relay the value of an iPSC bank in revealing common pathogenic mechanisms as well as differences in pathogenesis among patient subpopulations. As outlined above, numerous genetic aberrations are associated with the development of ALS/FTD. Some of the proteins encoded by causative genes can be categorized according to their distinct functional properties. For instance, TDP-43 and FUS both are involved in RNA metabolism. C9ORF72 and ubiquilin 2 (UBQLN2) both have a functional role in proteostasis. The proteins SOD1 and D-amino-acid oxidase (DAO) are associated with preventing oxidative stress. It therefore stands to reason that mutations in ALS-associated genes share underlying molecular pathways. Indeed, Kiskinis et al. (2014) have recently shown that patient derived iPSC MNs with genetic aberrations in* SOD1* and* C9ORF72* share a subset of molecular changes. Among these are transcriptional changes linked with elevated oxidative stress response, reduced mitochondrial function, and changes in cation channels and motor proteins [20]. A comprehensive iPSC library would allow evaluation of whether other disease-specific changes are observed across ALS cases with different causative genes, such as* SOD1* and* C9ORF72*. As an example, it has been shown that mutations in* SOD1* cause an impairment of neurofilament turnover [27]. This has been tested exclusively in* SOD1* MNs but not in other ALS/FTD genetic variants. An iPSC bank could aid confirmation of the presence of common pathogenic pathways and thus therapeutic targets with relevance to a broader patient population could be prioritised.

However, the fact that there are numerous causative genes with various mutations that result in ALS/FTD indicates that there is not one pathogenic mechanism underlying the disease, but rather there are multiple molecular changes that combine to cause overlapping phenotypes. For instance, more than 50 mutations in* TARDBP* have been associated with the pathogenesis of ALS to date [7]. Furthermore, there is different disease progression in patients carrying distinct* SOD1* mutations. Assessment of phenotypes in patients with* SOD1* mutations revealed that the missense mutation D90A, for example, causes a characteristic uniform phenotype with insidious onset, slowly ascending paresis beginning distally in the lower extremities, and long survival, whereas patients with the A4V mutation exhibit sudden onset and rapid disease progression [61]. Therefore although it is important to identify pathogenic mechanisms that can be targeted in a time and cost minimized manner with relevance to a broad patient population, this will not overcome the need to detect differences among distinct mutations in order to identify the most effective therapeutic target for individual patients.

### 3.3. Drug Screening Using Stem Cell Banks

Potential therapeutic compounds are developed based on their ability to interfere with an identified pathogenic mechanism. Drugs that significantly reverse disease phenotypes in models can then be further tested and chemically modified in order to have efficacy in humans. To date, there is only one drug on the market for ALS and its beneficial effects do not exceed 6 months [10]. Most other potential therapeutics have failed phase two and three clinical trials. An iPSC bank would serve as a source for screening potential drug candidates, predicting the effectiveness of compounds in terms of their toxicity, dosage, and individual patient response [58]. To successfully screen potential therapeutic compounds, a disease phenotype and a reliable screening assay must be established. For example, in ALS the disease phenotype classically includes increased protein aggregate formation and decreased survival rate of MNs, both of which can be evaluated in iPSC derived cultures.

As mentioned, a recent study using iPSCs derived from patients carrying mutations in either* SOD1* or* TARDBP* showed the therapeutic potential of the GSK-3b inhibitor kenpaullone by increasing iPSC derived MN survival [32]. Interestingly, kenpaullone showed higher efficacy than two compounds that failed in clinical trials (dexpramipexole and olesoxime), suggesting that the current system of drug development might be improved with the integration of iPSC screening. Another study focused only on* TARDBP* mutations in iPSC derived MNs for screening compounds and found that the histone acetyltransferase inhibitor anacardic acid ameliorates ALS-associated phenotype and is neuroprotective in MNs [35].

Once therapeutic effectiveness is established, compounds (e.g., kenpaullone) could be screened in various cell lines provided by an iPSC bank. This would enable assessment of effects on patient specific cells carrying different mutations, including cell lines from sporadic patients, and may subsequently pave the way for cost and time minimized drug discovery, with specific relevance to ALS/FTD patient subpopulations.

### 3.4. Challenges with iPSC Technology

Alongside issues mentioned such as inconsistent reprogramming among different research groups, there are various challenges with iPSC technology as it stands. Reprogramming efficiency is often low (less than 0.02% in fibroblasts [62]) resulting in a costly and time intensive process, often laborious in nature. An important aspect of iPSC reprogramming validity is the challenge of creating “footprint-free” models in which the resulting cell lines are free from genes involved in the reprogramming process. Various methods for the delivery of reprogramming genes have been developed, and the generation of footprint-free cell lines is becoming possible with vehicles such as episomal vectors and the Sendai virus. In both systems, the vectors remain episomal, not integrating into the host genome, and are subsequently abolished from reprogrammed cells [63, 64]. Reduced transfection efficiency due to induction of an antiviral response to viral vectors might further be avoided through development of synthetic mRNA to deliver the pluripotency genes [65]. Moreover the development of reprogramming protocols whereby reprogramming genes are replaced by small molecules is leading the way towards induction of pluripotency free from genetic manipulation [66–68]. Issues with collection of patient tissue samples may be reduced by the development of protocols that require samples with noninvasive collection strategies, and use of cell types that are easy to expand and maintain in cell culture will increase efficiency of the lengthy process. An issue particularly relevant to ALS/FTD is whether iPSC derived models adequately represent late onset diseases given that cell cultures are comparatively “young.” Attempts to tackle this issue include the introduction of age mimicking genes such as that which encodes the protein progerin, a truncated form of lamin A that is associated with premature ageing [69]. However relying on integration of genetic material is not ideal, and development of alternative methods for manipulation of cellular models would be desirable. Furthermore issues with abhorrent epigenetic silencing of genes as a result of reprogramming [70] and the notion that reprogramming causes changes in the genome, for example, through altering copy number variants or single nucleotide variants [71, 72], are being addressed. Despite the need to improve the reprogramming process and the various limitations with iPSC derived models, they remain an important tool, and when utilized alongside various other models they contribute to an increasingly robust research system.

## 4. Future Directions

The use of stem cells for disease modelling and drug screening offers great potential for discovery of effective treatments. However, no standardized protocols for reprogramming and differentiation exist among different research groups. Thus, there are often differences in ability to recapitulate disease phenotype in different laboratories. An iPSC bank that follows quality control and regulatory compliance may overcome these obstacles and give multiple research groups access to resources, as has been demonstrated by fibroblast banks for neurological diseases [73]. Initiatives such as HiPSCi and StemBANCC are working to establish comprehensive iPSC libraries. Once this is achieved, these banks might accelerate research and enable “clinical trial in a dish,” which could potentially reduce the time and cost of the currently lengthy process of drug discovery.

## Figures and Tables

**Table 1:** Genes associated with both ALS and FTD.

Gene	Locus	Protein	Putative function	Reference
*CHMP2B*	3p11	Charged multivesicular body protein 2B	Vesicle trafficking	[44]
*TARDBP*	1p36	TAR DNA binding protein 43	RNA metabolism	[39, 45, 46]
*VCP*	9p13	Valosine -containing protein	Ubiquitination; proteasomal degradation	[47]
*UBQLN2*	Xp11	Ubiquilin 2	Ubiquitination; proteasomal degradation	[48]
*C9ORF72*	9p21	Chromosome 9 open reading frame 72	Intracellular trafficking RNA metabolism	[49, 50]
*TUBA4A*	2q35	Tubulin, alpha 4A protein	Cytoskeleton dynamics	[51]

**Table 2:** Observations from stem cell derived models of known ALS/FTD genes.

Aberrant gene	Differentiated cell type	Main observations	Associated disease	Reference
*C9ORF72*	hiPSC derived neurons	RNA foci, RAN translation products, and increased susceptibility to cellular stress caused by autophagy inhibition	ALS/FTD	[22]
	hiPSC derived neurons	Specific susceptibility to glutamate excitotoxicity and mitigation of *C9ORF72* phenotype with antisense oligonucleotides therapeutics	ALS/FTD	[23]
	hiPSC derived MNs	Transcriptional changes and presence of RNA foci	ALS/FTD	[24]

*C9ORF72/TARDBP*	hiPSC derived MNs	Neuronal hyperexcitability followed by progressive loss of action potential output and synaptic activity	ALS/FTD	[52]

*PGRN*	hiPSC derived neurons	Increased sensitivity to kinase inhibition, reduced S6K2 levels, and neurite degeneration in absence of glia	FTD	[19]
	hiPSC derived neurons	Identification and validation of small molecules with therapeutic potential	FTD	[53]

*TARDBP*	hiPSC derived MNs	Mislocalization of TDP-43, decreased MN survival rate, and increased vulnerability to inhibition of the PI3K pathway	ALS/FTD	[17]
	hiPSC derived MNs	Identification of the histone acetyltransferase inhibitor anacardic acid as able to reverse disease phenotype	ALS/FTD	[35]
	hiPSC derived MNs	TDP-43 aggregation and feasibility of hiPSCs derived MNs for drug screening	ALS/FTD	[21]

*TARDBP/SOD1*	mESC derived MNs/hiPSCs derived MNs	Kenpaullone, a GSK3-inhibitor, increased survival of MNs more so than two potential therapeutic compounds that failed in clinical trials (olesoxime and dexpramipexole)	ALS	[32]

*SOD1*	hESC derived MNs/glia cells	MNs were shown to be selectively sensitive to toxic effects of cocultured *SOD1* glial cells	ALS	[30]
	hESC derived MNs/astrocytes	Selective MN toxicity correlates with increased inflammatory response in *SOD1* astrocytes	ALS	[31]
	hiPSC derived MNs	Identification of misregulated neurofilament	ALS	[27]

*SOD1/C9ORF72*	hiPSC derived MNs	Potential correlation between *SOD1* and *C9ORF72* pathogenesis, including elevated oxidative stress response	ALS	[20]

hiPSC = human induced pluripotent stem cell; mESC = mouse embryonic stem cell; hESC = human embryonic stem cell; MN = motor neuron.
